# Supplementary material for: Clinical Importance of Grading Tumor Spread through Air Spaces in Early-Stage Small-Lung Adenocarcinoma
Source: Cancers (Basel). 2024 Jun 14;16(12):2218. doi: 10.3390/cancers16122218 (PMC11201625; doi:10.3390/cancers16122218)
Supplement: Supplementary file 1 [file cancers-16-02218-s001.zip › cancers-3018180-supplementary.pdf]

Table S1. Demographic and preoperative characteristics of STAS (-), STAS (+)-L and STAS (+)-H groups. Comparison was performed using ANOVA for normal continuous variables and Kruskal-Wallis test for categorical ones.

Variables	STAS (-) N (%) mean±sd. (95% CI)	STAS (+)-L N (%) mean±sd. (95% CI)	STAS (+)-H N (%) mean±sd. (95% CI)	p-value	Total N (%) mean±sd. (95% CI)
**Number**	156	33	29		218
**Age**	65.2±9.95 (63.6-66.7)	65.7±8.49 (62.7-68.7)	66.1±11.44 (61.8-70.5)	0.879	65.4±9.92 (64.1-66.7)
				0.949	
**<65**	68 (43.6)	15 (45.5)	12 (41.4)		95 (43.6)
**≥65**	88 (56.4)	18 (54.5)	17 (58.6)		123 (56.4)
**Gender**				0.003	
**F**	97 (62.2)	23 (69.7)	9 (31.0)		129 (59.2)
**M**	59 (37.8)	10 (30.3)	20 (69.0)		89 (40.8)
**CCI**	3.2±1.70 (2.90-3.44)	3.4±1.84 (2.77-4.08)	3.3±2.10 (2.48-4.08)	0.751	3.2±1.77 (2.99-3.46)
				0.655	
**≤3**	96 (61.5)	18 (54.5)	19 (65.5)		133 (61.0)
**>3**	60 (38.5)	15 (45.5)	10 (34.5)		85 (39.0)
**Smoking history**				0.205	
**Never**	109 (69.9)	23 (69.7)	14 (48.3)		146 (67.0)
**Current**	21 (13.5)	5 (15.2)	4 (13.8)		30 (13.8)
**Ex≤15**	17 (10.9)	4 (12.1)	8 (27.6)		29 (13.3)
**Ex>15**	9 (5.8)	1 (3.0)	3 (10.3)		13 (6.0)
**PPY**	9.1±18.11 (6.18-11.91)	13.8±27.28 (4.09-23.43)	20.2±34.19 (7.15-33.16)	0.180	11.2±22.58 (8.22-14.25)
**Clinical staging**					
**T**				0.011	
**1a**	32 (20.5)	1 (3.0)	0 (0.0)		33 (15.1)
**1b**	41 (26.3)	14 (42.4)	10 (34.5)		65 (29.8)
**1c**	1 (0.6)	0 (0.0)	0 (0.0)		1 (0.5)

**2a**	80 (51.3)	17 (51.5)	19 (65.5)		116 (53.2)
**3**	2 (1.3)	1 (3.0)	0 (0.0)		3 (1.4)
**N**				0.600	
**0**	152 (97.4)	33 (100.0)	28 (96.6)		213 (97.7)
**1**	4 (2.6)	0 (0.0)	1 (3.4)		5 (2.3)
**Stages**				0.007	
**IA1**	31 (19.9)	0 (0.0)	0 (0.0)		31 (14.2)
**IA2**	42 (26.9)	15 (45.5)	9 (31.0)		66 (30.3)
**IA3**	1 (0.6)	0 (0.0)	0 (0.0)		1 (0.5)
**IB**	77 (49.4)	17 (51.5)	19 (65.5)		113 (51.8)
**IIA**	3 (1.9)	0 (0.0)	0 (0.0)		3 (1.4)
**IIB**	2 (1.3)	1 (3.0)	1 (3.4)		4 (1.8)
**CT findings**					
**Total tumor size (including GGO)**	17.2±4.13 (15.75-18.68)	17.2±4.13 (15.75-18.68)	17.8±3.36 (16.56-19.11)	0.806	17.5±3.77 (16.55-18.46)
**Consolidative size**	14.9±2.75 (13.97-15.91)	14.9±2.75 (13.97-15.91)	16.3±3.00 (15.12-17.40)	<0.001	15.6±2.92 (14.82-16.30)
**CT ratio**	0.9±0.18 (0.84-0.96)	0.9±0.18 (0.84-0.96)	0.9±0.12 (0.88-0.97)	<0.001	0.9±0.15 (0.87-0.95)
**0.5≥**	1 (3.0)	1 (3.0)	0 (0.0)		27 (12.4)
**0.5<**	32 (97.0)	32 (97.0)	29 (100.0)		191 (87.6)
**Pleural tagging**				0.437	
**N**	74 (47.4)	15 (45.5)	10 (34.5)		99 (45.4)
**Y**	82 (52.6)	18 (54.5)	19 (65.5)		119 (54.6)
**Location**				0.591	
**Medial**	19 (12.2)	6 (18.2)	4 (13.8)		29 (13.3)
**Lateral**	137 (87.8)	27 (81.8)	25 (86.2)		189 (86.7)
**mSUV value**	3.9±2.96 (1.75-5.92)	3.9 ± 3.0	3.2 ± 2.0	0.837	3.4±3.24 (2.65-4.07)

Table S2. Pathologic features of STAS (-), STAS (+)-L and STAS (+)-H groups. Comparison was performed using ANOVA for normal continuous variables and Kruskal-Wallis test for categorical ones.

Variables	STAS (-)<br>N (%)<br>mean±sd. (95% CI)	STAS (+)-L<br>N (%)<br>mean±sd. (95% CI)	STAS (+)-H<br>N (%)<br>mean±sd. (95% CI)	p-value	Total<br>N (%)<br>mean±sd. (95% CI)
**Predominant subtypes**				<0.001	
**lepidic**	42 (26.9)	1 (3.0)	0 (0.0)		43 (19.7)
**acinar**	102 (65.4)	27 (81.8)	19 (65.5)		148 (67.9)
**papillary**	4 (2.6)	1 (3.0)	6 (20.7)		11 (5.0)
**micropapillary**	0 (0.0)	1 (3.0)	0 (0.0)		1 (0.5)
**solid**	8 (5.1)	3 (9.1)	4 (13.8)		15 (6.9)
**Tumor size**					
**Including lepidic component**	16.6±3.89 (15.26-18.02)	16.6±3.89 (15.26-18.02)	17.1±3.23 (15.87-18.33)	0.794	16.7±4.43 (16.11-17.29)
**Invasive component**	15.1±2.66 (14.12-16.00)	15.1±2.66 (14.12-16.00)	16.0±2.70 (14.94-16.70)	<0.001	13.3±4.06 (12.74-13.82)
**invasive-tumor ratio**	0.93±0.137 (0.878-0.975)	0.93±0.137 (0.878-0.975)	0.94±0.989 (0.905-0.978)	<0.001	0.82±0.215 (0.789-0.846)
**Visceral pleural invasion (VPI)**				0.195	
**0**	73 (46.8)	16 (48.5)	11 (37.9)		100 (45.9)
**1**	82 (52.6)	15 (45.5)	18 (62.1)		115(52.8)
**2**	1 (0.6)	2 (6.1)	0 (0.0)		3 (1.4)
**Micropapillary pattern**				<0.001	
**No**	145 (92.9)	17 (51.5)	12 (41.4)		154 (70.6)
**Yes**	11 (7.1)	16 (48.5)	17 (58.6)		64 (29.4)
**Micropapillary pattern (%)**				<0.001	
**<10**	153 (98.1)	24 (72.7)	16 (55.2)		193 (88.5)
**≥10**	3 (1.9)	9 (27.3)	13 (44.8)		25 (11.5)
**Lymphovascular_invasion**				0.835	
**No**	150 (96.2)	32 (97.0)	29 (100.0)		211 (96.8)
**Yes**	6 (3.8)	1 (3.0)	0 (0.0)		7 (3.2)
**Perineural invasion**				1.000	
**No**	155 (99.4)	33 (100.0)	29 (100.0)		217 (99.5)
**Yes**	1 (0.6)	0 (0.0)	0 (0.0)		1 (0.5)
**Necrosis**				0.036	
**No**	151 (96.8)	31 (93.9)	25 (86.2)		207 (95.0)
**Yes**	5 (3.2)	2 (6.1)	4 (13.8)		11 (5.0)

**Pathologic stage**				0.001	
**IA1**	32 (20.5)	0 (0.0)	0 (0.0)		32 (14.7)
**IA2**	41 (26.3)	16 (48.5)	11 (37.9)		68 (31.2)
**IB**	83 (53.2)	17 (51.5)	18 (62.1)		118 (54.1)
**EGFR (+)**				0.015	
**No**	62 (39.7)	21 (63.6)	17 (58.6)		100 (45.9)
**Yes**	94 (60.3)	12 (36.4)	12 (41.4)		118 (54.1)
**Farthest distance of STAS**					
**Standard length scale (mm)**		1.0±0.20 (0.93-1.07)	2.9±0.71 (2.60-3.13)	<0.001	1.9±1.06 (1.60-2.14)
**Number of alveolar spaces**		4.4±1.46 (3.91-4.94)	10.3±4.12 (8.78-11.91)	<0.001	7.2±4.21 (6.12-8.29)

Table S3. Perioperative characteristics and comparison between STAS (-), STAS (+)-L and STAS (-)-L. Student t-tests were performed for normal continuous data and summarized with mean (±SD). Non-normal continuous variables are summarized with median [IQR or min, max] after performing chi-square tests. Kruskal-Wallis tests were used for categorical variables.

	Group						
Variables	STAS (-) (N=156)	STAS (+) (N=62)				p-value	Total (N=218)
		STAS (+)-L (N=33)	STAS (+)-H (N=29)	p-value	Total		
	N (%) mean±sd. (95% CI)	N (%) mean±sd. (95% CI)	N (%) mean±sd. (95% CI)		N (%) mean±sd. (95% CI)		N (%) mean±sd. (95% CI)
**Location of lobe**				0.559		0.866	
RUL	54 (34.6)	11 (33.3)	9 (31.0)		20 (32.3)		74 (33.9)
RML	12 (7.7)	4 (12.1)	2 (6.9)		6 (9.7)		18 (8.3)
RLL	29 (18.6)	8 (24.2)	5 (17.2)		13 (21.0)		42 (19.3)
LUL	41 (26.3)	7 (21.2)	6 (20.7)		13 (21.0)		54 (24.8)
LLL	20 (12.8)	3 (9.1)	7 (24.1)		10 (16.1)		30 (13.8)
**Type of resection**				0.631		0.588	
Wedge	26 (16.7)	3 (9.1)	5 (17.2)		8 (12.9)		34 (15.6)
Segmentectomy	12 (7.7)	4 (12.1)	3 (10.3)		7 (11.3)		19 (8.7)
Lobectomy	118 (75.6)	26 (78.8)	21 (72.4)		47 (75.8)		165 (75.7)
**PCNB**				0.317		0.070	
No	93 (59.6)	17 (51.5)	11 (37.9)		28 (45.2)		121 (55.5)
Yes	63 (40.0)	16 (48.5)	18 (62.1)		34 (54.8)		97 (44.5)

**Localization**				1.000		0.010	
**No**	136 (87.2)	32 (97.0)	29 (100.0)		61 (98.4)		197 (90.4)
**Yes**	20 (12.8)	1 (3.0)	0 (0.0)		1 (1.6)		21 (9.6)
**Frozen**				0.112		0.070	
**No**	78 (50.0)	18 (54.5)	22 (75.9)		40 (64.5)		118 (54.1)
**Yes**	78 (50.0)	15 (45.5)	7 (24.1)		22 (35.5)		100 (45.9)
**Types of surgery**						1.000	
**VATS**	153 (98.1)	32 (97.0)	29 (100.0)		61 (98.4)		214 (98.2)
**Open**	3 (1.9)	1 (3.0)	0 (0.0)		1 (1.6)		4 (1.8)
**Anesthesia time**				0.051		0.769	
	241.7±67.75 (230.97-252.39)	255.0±71.50 (229.65-280.35)	221.0±72.00 (193.65-248.42)		239.1±73.17 (220.53-257.69)		241.0±69.17 (231.72-250.18)
**Operation time**				0.013		0.585	
	176.4±62.30 (166.49-186.20)	194.6±63.41 (172.06-217.03)	158.45±69.22 (132.12-184.78)		177.7±68.11 (160.37-194.96)		176.7±63.85 (168.20-185.24)
**Adjuvant CTx.**				0.308		0.432	
**No**	129 (82.7)	28 (84.8)	27 (93.1)		55 (88.7)		184 (84.4)
**Yes**	27 (17.3)	5 (15.2)	2 (6.9)		7 (11.3)		34 (15.6)

Table S4. Recurrence rates of each group.

Recurrence	Group				Total
	STAS (-)	STAS (+)-L	STAS (+)-H	P-value	
Total	5 (3.2%)	3 (9.1%)	6 (20.7%)	0.010	14 (6.4%)
Locoregional recur	4 (2.6%)	2 (6.1%)	5 (17.2%)	0.004	11 (5.0%)
Distant recur	2 (1.3%)	2 (6.1%)	3 (10.3%)	0.024	7 (3.2%)
Locoregional + distance	0 (0.0%)	1 (3.0%)	2 (6.9%)	0.009	3 (1.4%)

Table S5. Recurrence rates of each group.

Recurrence	Group				Total
	STAS (-)	STAS (+)-L	STAS (+)-H	P-value	
Total	5 (3.2%)	3 (9.1%)	6 (20.7%)	0.010	14 (6.4%)
Locoregional recur	4 (2.6%)	2 (6.1%)	5 (17.2%)	0.004	11 (5.0%)
Distant recur	2 (1.3%)	2 (6.1%)	3 (10.3%)	0.024	7 (3.2%)
Locoregional + distance	0 (0.0%)	1 (3.0%)	2 (6.9%)	0.009	3 (1.4%)

Table S6. Overall and recurrence-free Survival rates.

		STAS (-)	STAS (+)-L	STAS (+)-H	p-value
Overall survival					0.630
	3-year (%)	100.0	100.0	89.1	
	5-year (%)	91.0	93.8	89.1	
Recurrence-free					<0.000
	3-year (%)	98.4	93.2	84.5	
	5-year (%)	96.3	93.2	52.8	

Table S7. Recurrence risk factor analysis. Cox-proportional hazard methods were used.

Variables	Univariate			Multivariate		
	p-value	Odds ratio	95% CI	p-value	Odds ratio	95% CI
Age	0.730	1.2	(0.389–3.858)			
Sex, male	0.166	0.4	(0.141–1.402)			
Consolidative size	0.034	1.1	(1.010–1.296)	0.608	1.1	(0.803–1.454)
Invasive tumor size	0.044	1.2	(1.004–1.339)	0.764	1.0	(0.757–1.227)
Micropapillary pattern	0.000	9.9	(2.753–35.470)	0.007	6.6	(1.688–26.116)
STAS (+) II	0.001	7.9	(2.470–25.551)	0.030	3.6	(1.132–11.470)
Alveolar spaces >3	0.013	4.3	(1.370–13.657)	0.734	0.7	(0.119–4.479)
Smoking history						
Ex >15	0.034	6.3	(1.145–34.179)	0.113	4.2	(0.711–24.973)

Abbreviations: CI, confidence interval; STAS, spread through air spaces
